# Base-pair resolution conservation data improves cell type specific sequence-to-expression prediction

**DOI:** 10.1093/bioinformatics/btag626

**Published:** 2026-08-21

**Authors:** Pooja Kathail, Forest Yang, Gabriel B Loeb, Nilah M Ioannidis

**Affiliations:** Center for Computational Biology, University of California, Berkeley, Berkeley, CA 94720, United States; Human Oncology and Pathogenesis Program, Memorial Sloan Kettering Cancer Center, New York, NY 10065, United States; Division of Nephrology, Department of Medicine, Institute for Human Genetics, Cardiovascular Research Institute, University of California, San Francisco, San Francisco, CA 94158, United States; Center for Computational Biology, University of California, Berkeley, Berkeley, CA 94720, United States; Department of Electrical Engineering and Computer Sciences, University of California, Berkeley, Berkeley, CA 94720, United States; Department of Applied Mathematics, University of California, Santa Cruz, Santa Cruz, CA 95064, United States; Biohub, San Francisco, CA 94158, United States

## Abstract

**Motivation:**

Genomic sequence-to-activity models can decipher gene regulatory mechanisms and predict the functional impact of regulatory variants. However, current models struggle to integrate information from sequences outside promoters, especially information from cell type specific regulatory elements.

**Results:**

Here, we propose incorporating base-pair resolution evolutionary conservation data into genomic sequence-to-expression predictors. We explore two training strategies—training from scratch or fine-tuning an existing sequence-only model with additional conservation input. We find that in both cases, base-pair resolution conservation data improves cell type specific sequence-to-expression prediction, with training from scratch yielding the greatest benefit. The improvement in cell type specific expression prediction can be attributed in part to the fact that models trained on sequence and conservation data learn to better recognize cell type specific regulatory elements than models trained on sequence alone.

**Availability:**

Code is available at https://github.com/ni-lab/basenji-phyloP.

## 1 Introduction

Genomic sequence-to-activity deep learning models can predict gene expression, chromatin accessibility, and transcription factor (TF) binding from DNA sequence (Sasse *et al.* 2024, Sokolova *et al.* 2024, Kathail *et al.* 2025). These models are promising tools to understand gene regulation and the impact of non-coding genetic variants. However, current models struggle to capture the effects of enhancers (Karollus *et al.* 2023), particularly cell type specific enhancers (Kathail *et al.* 2024), limiting their performance when modeling gene expression. 47% of candidate *cis*-regulatory elements (cCREs) identified by the ENCODE project are highly conserved (Andrews *et al.* 2023), and conserved genomic positions are highly enriched for complex disease heritability (Sullivan *et al.* 2023). Moreover, prior work has demonstrated the utility of multi-species data for training sequence-based deep learning models (Kelley 2020, Li *et al.* 2023, Benegas *et al.* 2025). Motivated by these observations, we sought to understand whether incorporating additional sources of data—namely, evolutionary conservation data—would enable models to better identify functional sequence elements, such as cell type specific enhancers.

To explore the utility of incorporating evolutionary conservation and acceleration to improve sequence-to-expression prediction, we used phyloP, which measures sequence conservation and acceleration at base-pair resolution (Pollard *et al.* 2010, Zoonomia Consortium 2020). We incorporated this additional information into the Basenji2 modeling framework, a convolutional neural network that makes chromatin accessibility, TF binding, histone modification, and gene expression predictions using a ∼55 kb receptive field (Kelley 2020). We employ the Basenji2 architecture to explore modelling choices for incorporating phyloP because it is relatively efficient to train compared to more recent, larger models, allowing for more rapid iteration. We find that incorporating base-pair resolution conservation data improves cell type specific sequence-to-expression prediction over the baseline Basenji2 model. We find that conservation data is most valuable for improving predictions for constrained genes and genes with more local regulatory elements. To understand the reasons for this improvement, we interrogated model attributions and found that models trained on sequence and conservation data learn the effects of cell type specific regulatory elements better than models trained on sequence alone.

## 2 Methods

### 2.1 Training data

We obtained processed training, validation, and test data used to train Basenji2 from Kelley (2020) and base-pair resolution phyloP scores from the Zoonomia project (Zoonomia Consortium 2020). We created a new “Sequence + phyloP” version of the Basenji2 data by adding an additional channel to each Basenji2 sequence containing base-pair resolution phyloP scores. Bases without phyloP scores were set to the mean genome-wide phyloP score.

### 2.2 Model architecture and training

We trained models based on the Basenji2 architecture and hyperparameters (Kelley 2020), using a modified version of the Basenji training pipeline. Models trained from scratch were trained for a maximum of 75 epochs with a patience of 30 epochs on both the human and mouse data, followed by a subsequent training phase with a patience of 10 epochs on only the human data.

For the fine-tuning approach, we initialized all model parameters with weights from the published Basenji2 model (Kelley 2020), with the exception of the final channel of the first convolutional layer, which corresponds to the newly added phyloP channel of the input sequence, and was randomly initialized. For the first training step, we froze all model weights except the newly added, randomly initialized weights. We then unfroze all model weights and fine-tuned on the sequence + phyloP data for 20 epochs.

### 2.3 Cell type specific gene expression prediction evaluation

We employed two different sets of metrics to evaluate cell type specific gene expression prediction. First, similar to the approach in Zhou *et al.* (2018), we quantified the correlation (Pearson *R* and Spearman *R*) between predicted and observed cell type specific gene expression profiles. Specifically, for each CAGE track, we correlated the log(fold change) over cell type average of the predictions with the log(fold change) over cell type average of the observed gene expression values. We refer to this metric as “logFC over cell type average.” Second, similar to the approach in Avsec *et al.* (2021), at each gene TSS we quantified the correlation (Pearson *R* and Spearman *R*) between predicted and observed gene expression across CAGE tracks. We refer to this metric as “Correlation across tracks.” In both cases, we restricted our evaluation to CAGE tracks and annotated gene TSSes.

### 2.4 Attribution analysis

To compute model attributions, we used DeepSHAP as implemented in the shap (Lundberg and Lee 2017) package, which is equivalent to averaging the results of running DeepLIFT (Shrikumar *et al.* 2017) over multiple references. For references, we used 32 non-TSS-containing sequences to ensure low CAGE expression. DeepLIFT performs a backpropagation algorithm to compute node attributions at each layer, which represent the contribution of each node to the model’s prediction on a query input, with respect to the reference. The attributions at the input layer are returned as the final attributions and are available per-base-pair. DeepLIFT attributions satisfy the summation-to-delta property: the sum of attributions across all features equals the difference between the query and reference prediction. Attribution plots depict these unscaled attributions. For each sequence, the model predicts CAGE expression across 896 bins covering 114 688 base-pairs, for 638 CAGE experiments (tracks) in addition to other tracks. Since DeepLIFT requires a scalar output, we used the sum of CAGE values in the bin containing the TSS plus the two flanking bins, for a given CAGE track or the mean of all CAGE tracks. Positive/negative attributions correspond to potential enhancing/repressing parts of the sequence.

To calculate attributions contained in cCREs, each set of cCREs was overlapped with sequences in the Basenji2 dataset, and attributions were computed with respect to each TSS in each sequence. Each data point represents a different TSS and attributions within all cCREs in the sequence containing that TSS. For each cCRE set, we computed attributions with respect to CAGE measured in the corresponding cell type. To compute attribution fractions, we summed attributions in cCREs, restricting to positions at least 1 kb away from the TSS, and divided by the sum of attributions at least 1 kb away from the TSS. Excluding attributions within 1 kb of the TSS was done in order to highlight distal regulatory elements. To compute the attribution fraction in cCREs within a particular distance bin, e.g. 5 kb-10kb, for each sequence, we computed attribution fraction in cCREs whose distance to the TSS (by closest endpoint) fell within that range.

### 2.5 eQTL classification

We obtained GTEx v8 eQTLs fine-mapped using the SuSiE method (Wang *et al.* 2020) from the Enformer Supplementary Data (Avsec *et al.* 2021, Wang *et al.* 2021). We selected six tissues that had large sample sizes and well-matched CAGE tracks to use for our evaluation: Adipose subcutaneous, Artery tibial, Cells cultured fibroblasts, Skin sun exposed lower leg, Testis, and Thyroid. To compute variant effect predictions, we subtracted the reference allele prediction from the alternate allele prediction across the central three 128 bp prediction bins centered at the variant. We used variant effect predictions from the tissue-matched CAGE track to compute the eQTL classification AUROC.

## 3 Results

### 3.1 Evolutionary conservation data can prioritize candidate *cis*-regulatory elements

**Figure 1:**
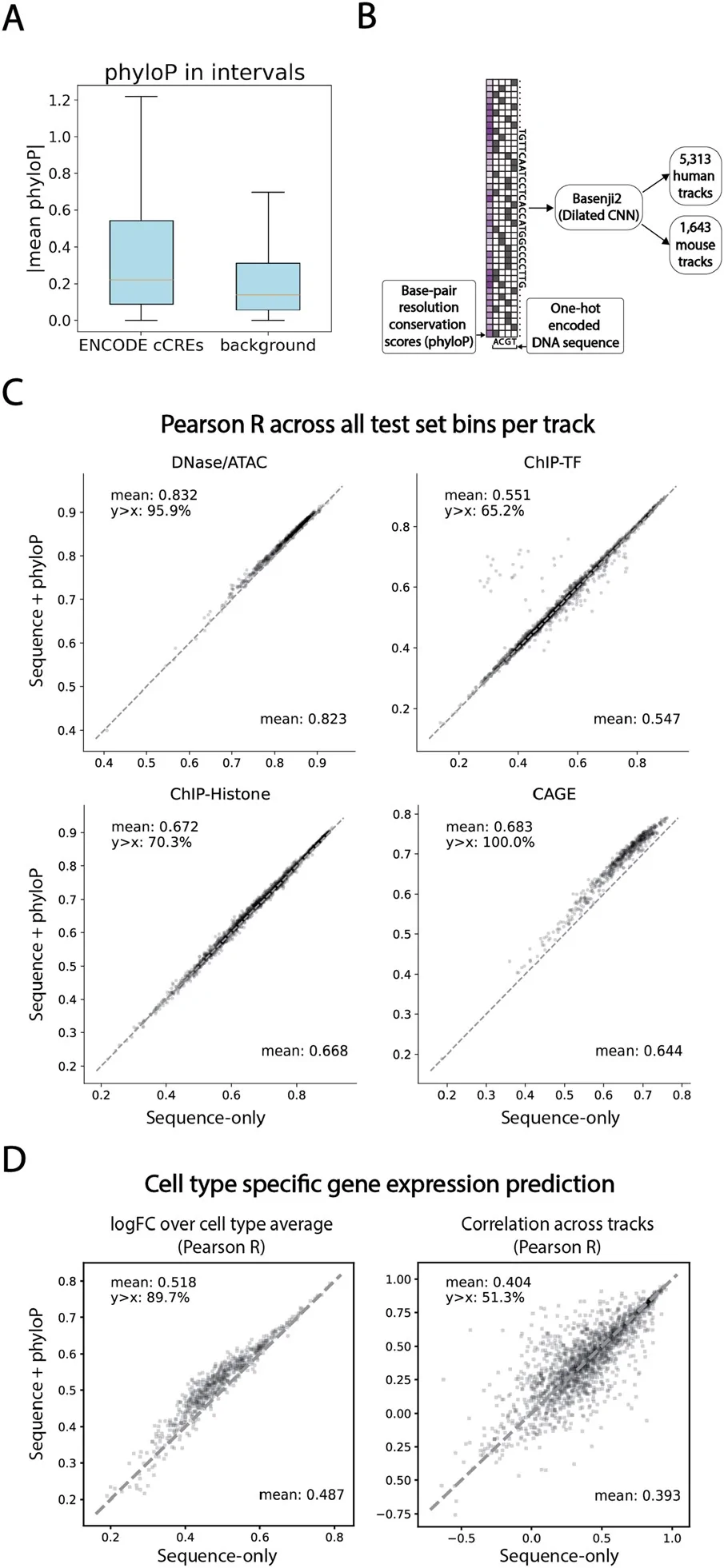
Utilizing base-pair resolution conservation scores to improve genomic sequence-to-activity models. (A) ENCODE cCREs are more highly conserved than random genomic intervals (one-sided Mann–Whitney *U* test, *P* < 1e − 48). (B) Model diagram illustrating how base-pair resolution conservation scores (phyloP) are incorporated into the Basenji2 model as an additional input channel. A description of the datasets used to train and evaluate the models presented in this manuscript is provided in Table S1. (C) Comparison of a model trained with base-pair level phyloP scores (“Sequence + phyloP,” *y*-axis) to Basenji2 (Kelley 2020) (“Sequence-only,” *x*-axis) across four assay types. Each point corresponds to a different output track in the human data and performance is reported as the Pearson correlation computed across all 128 bp bins in the test set. Full results for multiple model replicates and fine-tuned models are shown in Table 1. (D) Comparison of a “Sequence + phyloP” model (*y*-axis) to Basenji2 (Kelley 2020) (“Sequence-only,” *x*-axis) for cell type specific gene expression prediction. We use two metrics to evaluate cell type specific performance. In the left plot, each point corresponds to a different CAGE track. Measured and predicted gene expression values are normalized by a log(fold change) over the average across CAGE experiments before computing the Pearson correlation across genes. In the right plot, we show the Pearson correlation across CAGE tracks for each gene. Here, each point corresponds to a different gene and values for each CAGE track were standardized to have zero mean and variance of 1 across genes. Full results for multiple model replicates and fine-tuned models are shown in Table 1.

We first sought to verify the utility of evolutionary conservation data for prioritizing CREs. To do so, we quantified the absolute mean Zoonomia phyloP score in randomly sampled ENCODE cCREs and random genomic intervals matched for size, TSS distance, and GC content (Figs. 1A, S1) (ENCODE Project Consortium 2012). As expected, we found that ENCODE cCREs have higher absolute phyloP scores than random genomic intervals, suggesting that evolutionary conservation data may be useful for prioritizing regulatory regions and functional motifs.

### 3.2 Base-pair resolution evolutionary conservation data improves cell type specific sequence-to-expression prediction

To investigate the utility of evolutionary conservation data for improving sequence-to-expression prediction, we incorporated base-pair level phyloP scores into the Basenji2 model architecture. Basenji2 takes one-hot encoded DNA sequences as input and predicts chromatin accessibility, TF binding, histone modification, and gene expression across 5313 human and 1643 mouse tracks. A description of the datasets used to train and evaluate the models presented in this manuscript is provided in Table S1.

We chose to incorporate phyloP as an additional channel to each input sequence (Fig. 1B). In early experiments, we also evaluated additional methods for incorporating phyloP—by using phyloP to derive base-pair level importance scores that were then used to multiplicatively scale early convolutional outputs—but this approach performed no better than simply incorporating phyloP as an additional input channel (Table S2). Therefore, we used the simple approach of adding a phyloP input channel in all subsequent results. We reasoned that incorporating phyloP could allow the model to use the conservation information to learn which bases within each sequence may be functionally important, effectively serving as a prior on the functional importance of each base. We explored two training strategies: (i) training from scratch and (ii) finetuning the existing Basenji2 sequence-only model. For the second approach, we initialized all model parameters with weights from the published Basenji2 model (Kelley 2020), with the exception of the final channel of the first convolutional layer, which corresponds to the newly added phyloP channel of the input sequence and was randomly initialized (Methods). We refer to models trained from scratch as “Sequence + phyloP” and models trained using the finetuning approach as “Sequence + phyloP finetuned.”

The performance of both training strategies compared to sequence-only Basenji models is shown in Table 1. We first report the test set performance—as measured by the Pearson correlation between predictions and experimental measurements across all 128 bp genomic bins in the test set (“Pearson correlation across all test set bins”)—for each assay type predicted by Basenji2 (Table 1 and Fig. 1C). Across all assay types, both training strategies that make use of conservation data outperform models trained on sequence alone, with the largest improvement for CAGE tracks. For CAGE gene expression tracks, we also report the Pearson correlation across the subset of test set bins that contain a gene TSS [“CAGE (gene TSSs)”] (Table 1). Finally, we report how well models predict cell type specific differences in gene expression [“Cell type specific gene expression prediction (gene TSSs)”], a more challenging but biologically important problem (Table 1 and Fig. 1D). Both training strategies that make use of conservation data outperform models trained on sequence alone for predicting cell type specific differences in gene expression. Interestingly, models trained from scratch yield better cell type specific expression predictions than fine-tuned models.

**Table 1:** Prediction performance of sequence-only and sequence + phyloP models trained on cross-species human and mouse data.

Model	**Pearson correlation across all test set bins**					**Cell type specific gene expression prediction (gene TSSs)**			
	DNase/ATAC	ChIP (TF)	ChIP (Histone)	CAGE (all bins)	CAGE (gene TSSs)	logFC over cell type average (Pearson R)	logFC over cell type average (Spearman R)	Correlation across tracks (Pearson R)	Correlation across tracks (Spearman R)
Sequence-only (Kelley 2020)	**0.823**	**0.547**	**0.668**	**0.644**	**0.770**	**0.487**	**0.472**	**0.393**	**0.392**
Sequence-only (retrained)	0.818 ± 0.0003	0.542 ± 0.0008	0.665 ± 0.0013	0.644 ± 0.0029	0.759 ± 0.0005	0.478 ± 0.0017	0.465 ± 0.0009	0.381 ± 0.0017	0.379 ± 0.0025
Sequence + phyloP	0.831 ± 0.0018 (*)	0.550 ± 0.0016 (*)	0.671 ± 0.0023	0.681 ± 0.005 (*)	0.773 ± 0.0025 (*)	0.522 ± 0.0052 (*)	0.499 ± 0.0029 (*)	0.406 ± 0.0028 (*)	0.398 ± 0.0039 (*)
Sequence + phyloP finetuned	0.829 ± 0.0008 (*)	0.551 ± 0.0004 (*)	0.674 ± 0.0003 (*)	0.657 ± 0.0012 (*)	0.776 ± 0.0019 (*)	0.506 ± 0.0028 (*)	0.486 ± 0.0033 (*)	0.397 ± 0.0014 (*)	0.390 ± 0.0009 (*)
Enformer	0.819	0.550	0.656	0.692	0.800	0.587	0.561	0.481	0.477

Where possible, we report standard deviations over three model replicates. The highest average correlation in each column among the Basenji2-based models (including the sequence-only and sequence + phyloP models) is indicated in bold. (*) indicate sequence + phyloP models with significantly higher prediction performance (*P* < 0.01) based on *P* values from a *t*-test against “Sequence-only (retrained),” corrected for multiple testing with Benjamini-Hochberg. Cell type specific gene expression prediction metrics are described in Methods.

We also report the performance of the larger Enformer (Avsec *et al.* 2021) model on the metrics described above (Table 1). Enformer improves upon the Basenji2 model by incorporating two key features: a larger receptive field (∼200 kb rather than ∼55 kb), and a higher capacity, more expressive transformer architecture. Our sequence + phyloP model has neither of these features. Therefore, we performed a comparison with Enformer in order to understand how leveraging a new type of information in genomic sequence models—evolutionary conservation—can complement other existing avenues for model improvement. We found that on several metrics—DNase/ATAC, TF ChIP-seq, and histone-ChIP-seq prediction—the sequence + phyloP model matches or exceeds the performance of Enformer (Table 1). On other metrics, such as the Pearson correlation in logFC gene expression, the sequence + phyloP model meaningfully reduces the gap between Basenji2 and Enformer. The sequence + phyloP model is also able to be trained using a small fraction of the compute needed for Enformer, indicating that evolutionary conservation data can potentially augment existing avenues for model improvement.

Next, we evaluated whether models were making use of the base-pair resolution phyloP scores to learn about important functional bases, or simply better recognizing conserved regulatory regions. In particular, we performed an ablation analysis where we trained sequence + phyloP models from scratch after smoothing the phyloP data to 10 bp or 100 bp resolution (Table 2). We found that base pair and 10 bp resolution data perform similarly, while 100 bp resolution performs worse. On many metrics, the model trained with 100 bp smoothing yielded little to no improvement compared to a baseline sequence-only model. These results suggest that relatively granular conservation data, at base-pair or motif resolution, provides utility beyond what can be gained from identification of conserved regulatory regions.

**Table 2:** Prediction performance of models trained with varying resolutions of conservation data.

Model	**Pearson correlation across all test set bins**					**Cell type specific gene expression prediction (gene TSSs)**			
	DNase/ATAC	ChIP (TF)	ChIP (Histone)	CAGE (all bins)	CAGE (gene TSSs)	logFC over cell type average (Pearson R)	logFC over cell type average (Spearman R)	Correlation across tracks (Pearson R)	Correlation across tracks (Spearman R)
Sequence-only (Kelley 2020)	**0.823**	**0.547**	**0.668**	**0.644**	**0.770**	**0.487**	**0.472**	**0.393**	**0.392**
Sequence-only (retrained)	0.818 ± 0.0003	0.542 ± 0.0008	0.665 ± 0.0013	0.644 ± 0.0029	0.759 ± 0.0005	0.478 ± 0.0017	0.465 ± 0.0009	0.381 ± 0.0017	0.379 ± 0.0025
Sequence + phyloP (base-pair resolution)	0.831 ± 0.0018 (*)	0.550 ± 0.0016 (*)	0.671 ± 0.0023	0.681 ± 0.0050 (*)	0.773 ± 0.0025 (*)	0.522 ± 0.0052 (*)	0.499 ± 0.0029 (*)	0.406 ± 0.0028 (*)	0.398 ± 0.0039 (*)
Sequence + phyloP (10bp smoothing)	0.831 ± 0.0006 (*)	0.551 ± 0.0011 (*)	0.673 ± 0.0007 (*)	0.675 ± 0.0049 (*).	0.775 ± 0.0029 (*)	0.523 ± 0.0021 (*)	0.502 ± 0.0012 (*)	0.409 ± 0.0012 (*)	0.401 ± 0.0024 (*)
Sequence + phyloP (100 bp smoothing)	0.824 ± 0.0022	0.544 ± 0.0046	0.666 ± 0.0027	0.652 ± 0.0101	0.761 ± 0.0041	0.5 ± 0.0104	0.481 ± 0.0064	0.394 ± 0.0034	0.387 ± 0.0024

Where possible, we report standard deviations over three model replicates. The highest average correlation in each column is indicated in bold. (*) indicate sequence + phyloP models with significantly higher prediction performance (*P* < 0.01) based on *P* values from a *t*-test against “Sequence-only (retrained),” corrected for multiple testing with Benjamini-Hochberg. Cell type specific gene expression prediction metrics are described in Methods.

### 3.3 Conservation data improves learning of cell type specific regulatory elements

We hypothesized that improved ascertainment of functional regulatory elements underlies improved gene expression predictions by models trained with conservation data. Sequence-to-expression models struggle to identify distal, cell type specific enhancers (Karollus *et al.* 2023). Conservation tracks could potentially contribute to ascertainment of conserved regulatory elements directly at inference time, or indirectly contribute to ascertainment of regulatory elements by helping models learn regulatory element sequence motifs during model training.

**Figure 2:**
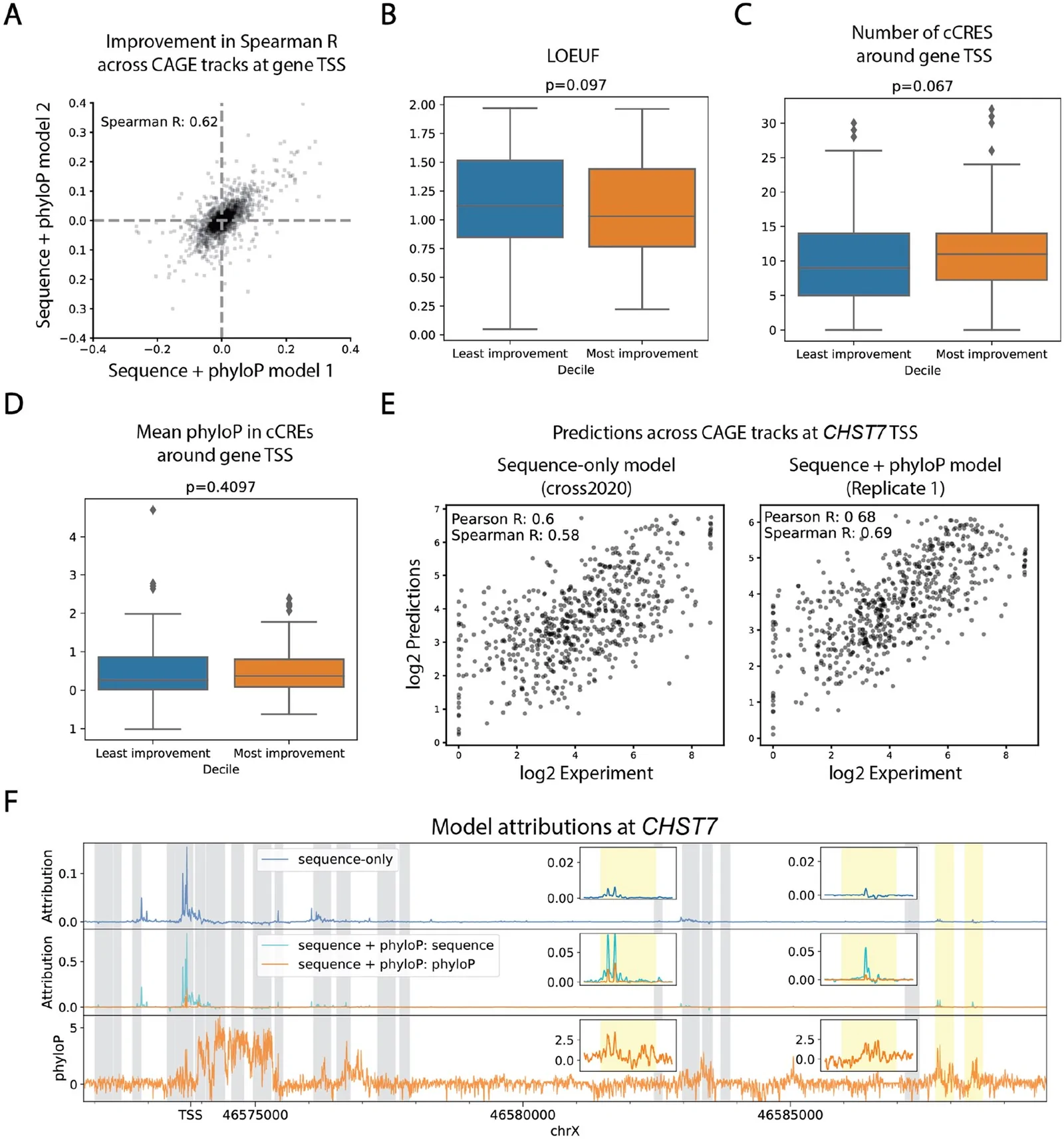
Models trained with conservation data better predict expression for constrained genes and genes with many nearby regulatory elements. (A) Models trained with conservation data consistently improve cell type specific expression predictions for the same genes. For two replicate sequence + phyloP models, the improvement relative to Basenji2 in the Spearman correlation across CAGE tracks at gene TSSs is shown. (B–D) Boxplots comparing several gene properties for the top and bottom decile of genes with the highest and lowest improvement in Spearman R across tracks when using sequence + phyloP models. *P* values are from a two-sided Mann–Whitney *U* test. The most improved genes are more constrained (lower LOEUF (Karczewski *et al*. 2020)) (B) and have more nearby cCREs (10 kb window around the gene TSS) (C), but show no trend in mean phyloP within nearby cCREs (10 kb window around the gene TSS) (D). (E) Scatterplots comparing experimentally measured and predicted CAGE gene expression values for *CHST7* (left: sequence-only model, right: sequence + phyloP model). Each dot represents expression at the *CHST7* TSS for a different CAGE track. (F) 10-bp window smoothed sequence-only model attributions, sequence + phyloP model attributions, and phyloP scores within a 24 kb locus surrounding the *CHST7* TSS (chrX:46565790–46589789). Sequence + phyloP model attributions are split into the contribution from each input modality (sequence and phyloP). ENCODE cCREs are highlighted in gray. Highlighted in yellow are ENCODE cCREs which overlap peaks in sequence + phyloP model attributions that are not present or much weaker in sequence-only model attributions. Insets show yellow highlighted regions, plus 100 base-pairs on each side, in order from left to right. The maximum *y*-axis value of each inset is set to the highest value in the corresponding row of the plot >1 kb away from the TSS.

We first evaluated whether the improvements in cell type specific predictions that we observed for sequence + phyloP models were consistent for individual genes across independently trained models. We observed reproducible effects of incorporating conservation on cell type specific gene expression prediction (Fig. 2A). We therefore sought to understand what gene features contribute to improved predictions by sequence + phyloP models.

We found several gene properties that distinguish genes with the highest and lowest improvement in cell type specific prediction with sequence + phyloP models. In particular, we found that the most improved genes are more constrained [lower LOEUF (Karczewski *et al.* 2020)] and have more nearby cCREs, indicating that conservation data may be helping learn complex gene regulatory logic of these constrained genes (Fig. 2B and C). In addition, we found that the most improved genes are more highly expressed and have a lower coefficient of variation across cell types (Fig. S2A and B). We speculate that the greater improvement by conservation incorporating models for more highly expressed and less variable genes may be due to reduced measurement error for these genes.

We were also curious whether conservation signal distinguishes the genes with the highest and lowest improvement in cell type specific prediction with sequence + phyloP models. We found that the mean phyloP in cCREs in a 10 kb region around the gene TSS is not significantly different between the top and bottom deciles of genes (Fig. 2E). We observed similar findings when we considered both conservation and acceleration signal by looking at absolute phyloP scores, or expanded to considering phyloP in the full 10 kb region around the gene TSS (Fig. S2C and D).

We examined *CHST7*, a gene for which the sequence + phyloP models better predict cell type specific expression (Fig. 2E). Examination of model attributions at the *CHST7* locus revealed two distal sequence elements lying 14 kb from the TSS at which sequence + phyloP models have dramatically higher attributions than sequence-only models (Fig. 2E). Both of these sequence elements are annotated by ENCODE as cCREs, suggesting that attributions at these regulatory elements may contribute to the improved gene expression predictions for *CHST7* by sequence + phyloP models.

To further interrogate how conservation may improve gene expression prediction at *CHST7*, we examined the conservation of the two cCREs at which the sequence + phyloP models had higher attributions. In both cases, we observed that the regions with higher attributions by the sequence + phyloP models were conserved (Fig. 2E). Higher model attribution at conserved elements may be due to a direct effect of phyloP on model predictions at inference time or an indirect effect of phyloP contributing to learning of sequence motifs at conserved elements during model training. To distinguish between these possibilities, we separated the attributions of the sequence + phyloP models into those coming from the phyloP channel versus the sequence channels (Fig. 2E). We observed that the increased attribution at these two conserved cCREs was primarily driven by higher sequence attributions, with a smaller contribution derived directly from the phyloP channel. These observations suggest that at least at these conserved regulatory elements, the major contribution of phyloP is to affect model ascertainment of sequence motifs.

To more systematically test the hypothesis that conservation is primarily influencing ascertainment of sequence motifs during training rather than directly influencing predictions at individual conserved regulatory elements, we examined the contribution of phyloP to gene expression predictions across genes for the sequence + phyloP models. Across genes we observed that the contribution of phyloP is about one-fifth of the contribution of sequence to the sequence + phyloP model’s predictions (Fig. S3). Together with the phyloP smoothing experiments, these observations suggest that base-pair level conservation data may improve predictions both through directly increasing attributions at conserved regulatory elements and by helping models learn functional sequence motifs located within regulatory elements.

For multiple other genes whose predictions are improved by phyloP, we also observed conserved cCREs with noticeably higher sequence + phyloP model attributions than sequence-only model attributions (Fig. S4), in contrast to genes where predictions are worsened (Fig. S5), which also show fewer neighboring cCREs overall. To test the hypothesis that improved ascertainment of these conserved regulatory elements explains improved cell type specific prediction for these genes, we reevaluated models after ablating the indicated regions. As expected, ablating these regions often had a larger effect on predicted expression values for the sequence + phyloP models than for the sequence-only models (Fig. S6A). For one of the evaluated genes, *FAM43A*, ablation of a single conserved cCRE was sufficient to reduce cell type specific expression prediction by the sequence + phyloP model to the level observed by the sequence only model. In contrast, for four other evaluated genes, ablation of 1 or 2 prominent conserved cCREs was not sufficient to reduce cell type specific prediction by sequence + phyloP models to the level observed in sequence-only models (Fig. S6B). Together these results suggest that training with conservation can improve model ascertainment of regulatory elements and that improvements in cell type specific gene expression are often distributed across attributions at multiple regulatory elements.

**Figure 3:**
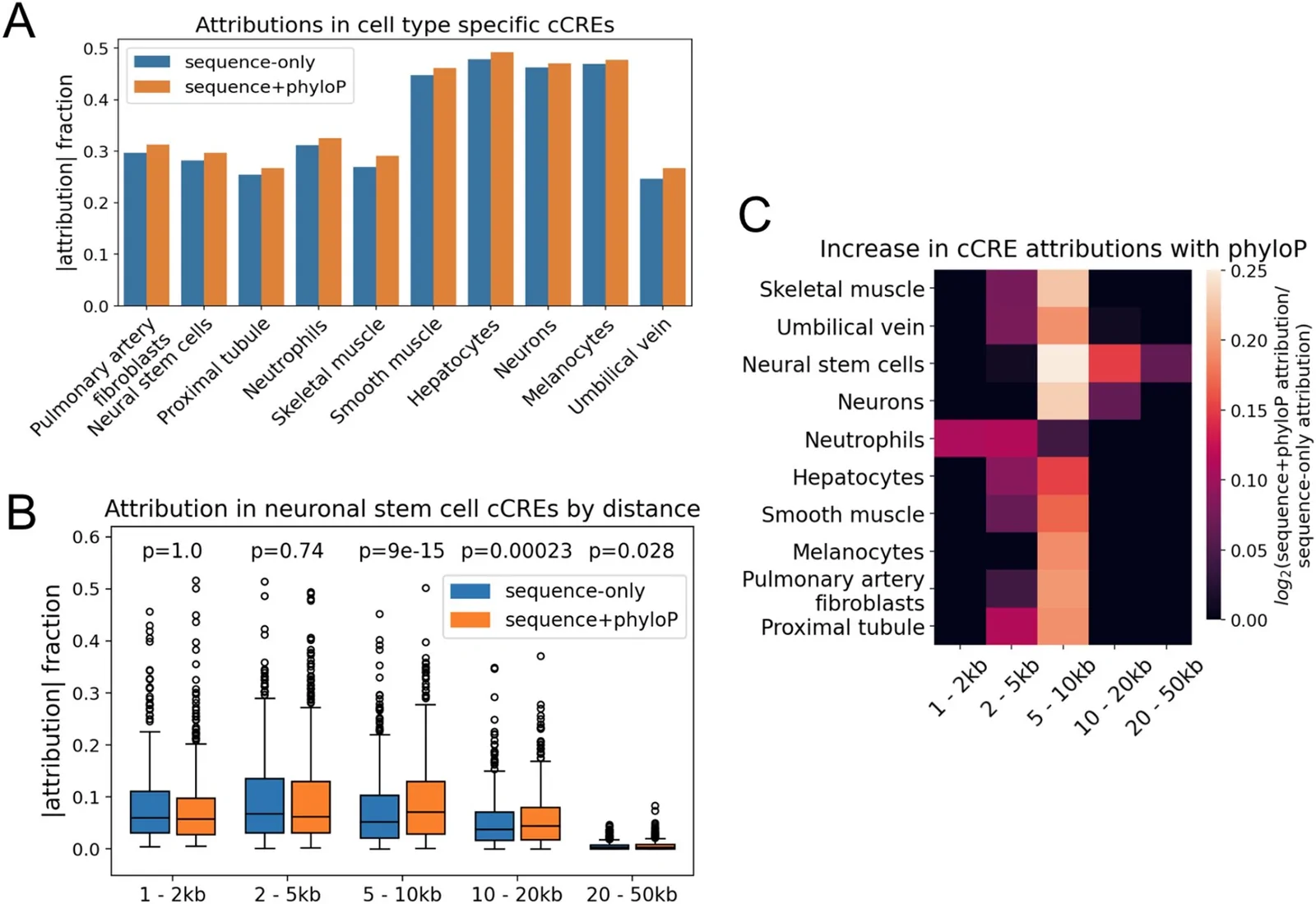
Models trained with conservation data better learn the effects of cell type specific regulatory elements. (A) Comparison of median absolute CAGE attribution fraction contained within cell type specific ENCODE cCREs for a sequence-only and a sequence + phyloP model. Attributions were computed separately for CAGE signal in each cell type. Attribution fraction was computed per sequence and TSS, restricted to positions at least 1 kb away from the TSS (Methods). The sequence + phyloP model has higher cCRE attributions across all 10 cell types (one-sided Binomial test, *P *= 0.001). The absolute value was taken because a cCRE may be activating or repressing. (B) Neural stem cell CAGE attribution fraction in neural stem cell cCREs, stratified by cCRE distance (Methods). *P* values are from the one-sided Wilcoxon signed-rank test. (C) Distance-stratified log fold-changes of the sequence + phyloP model vs. sequence-only model attribution fraction in cCREs for each cell type.

Next, we evaluated whether the addition of conservation helped the model learn cell type specific regulatory elements. To this end, we computed the fraction of model attributions within ENCODE cCREs (ENCODE Project Consortium 2012) from 10 different cell types (Methods). Specifically, we evaluated the contribution of these elements to model predictions relative to all non-promoter sequence (>1 kb from TSS) (Fig. 3). Comparing a sequence + phyloP and a sequence-only model, we observed consistently higher attribution fraction in cCREs for every cell type (Fig. 3A). This suggests that incorporating conservation information helps models identify cell type specific regulatory elements. Given the challenge of ascertaining distal regulatory elements, we were interested in quantifying where phyloP was improving attributions in distal cCREs. In neuronal stem cells, we observed significant increases in attributions at cCREs from 5–50 kb, but no improvement at more proximal cCREs (Fig. 3B). To extend this observation we examined attributions within cCREs in 10 cell types and observed that in all but one cell type (neutrophils), models trained with phyloP had the largest increase in attributions at cCREs between 5–10 kb, with increased attributions at cCREs between 2–5 kb and 10–20 kb in some cell types (Fig. 3C). Together these data indicate that the addition of phyloP increases model attributions to cell type specific cCREs at moderate distances to the TSS (typically 2–20 kb). Interestingly, we observed that the distances at which the sequence + phyloP models increased attributions in neutrophils was shifted much closer to the TSS than in other cell types (Fig. 3C). This observation is consistent with data that neutrophil progenitors dramatically disrupt long range chromatin interactions by downregulating components of the cohesin complex to promote nuclear deformability and migration (Patta *et al.* 2024).

**Figure 4:**
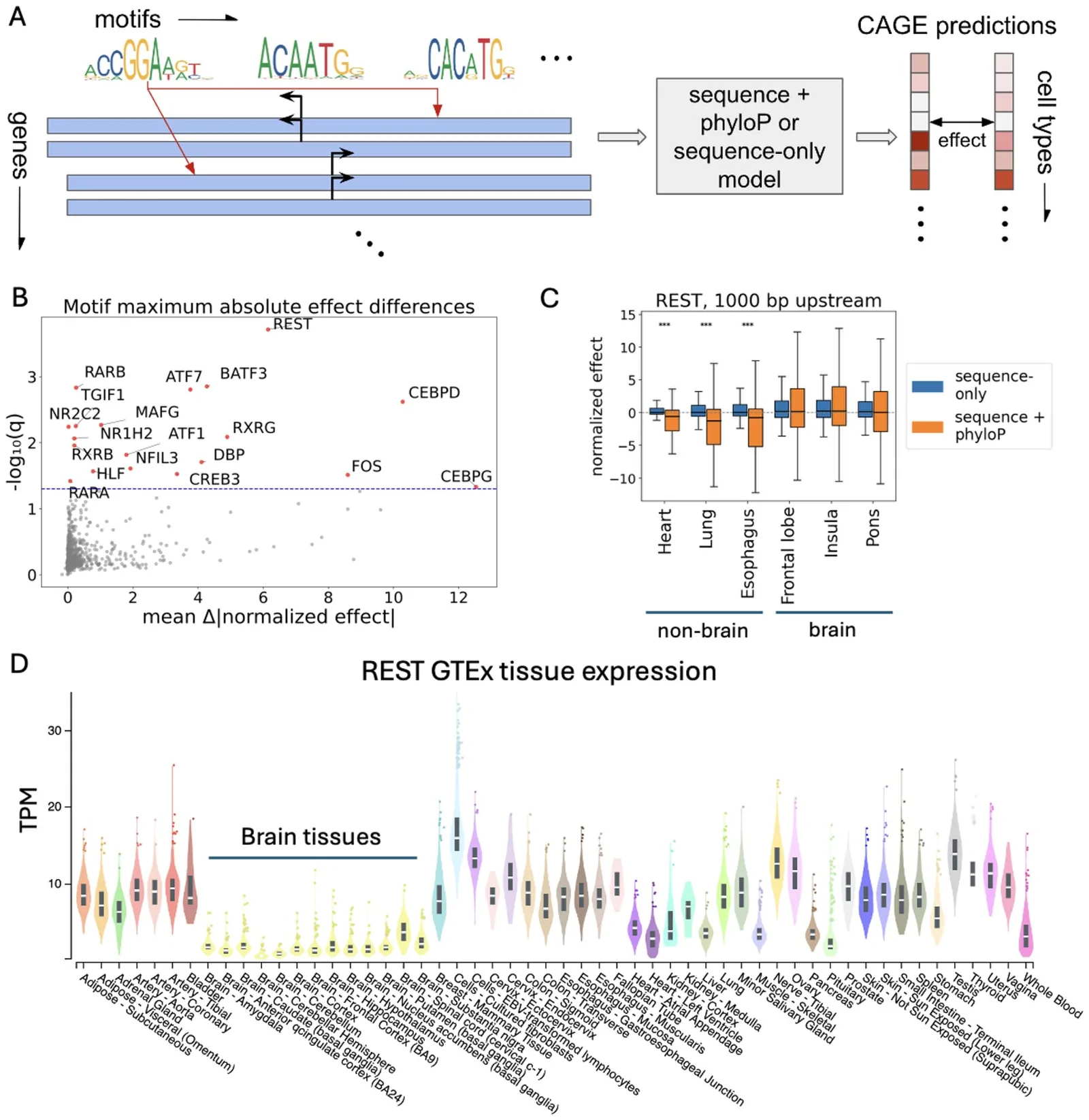
Motif insertion highlights differential learning of REST motif. (A) Schematic of the motif insertion analysis. Each motif in a motif collection (Weirauch *et al*. 2014) is inserted *in silico* at several positions (100, 1000, and 10000 bp upstream) relative to the TSS for 100 genes. The effect of the inserted motif is defined as predicted expression after insertion minus predicted expression before insertion. (B) Volcano plot showing, for each motif, whether it has a significantly higher absolute effect in the sequence + phyloP model compared to the sequence-only model in any context (cell type and insertion distance), by choosing the context with the greatest Wilcoxon-signed rank statistic for each motif. The *x*-axis is the mean difference in absolute effect (sequence + phyloP minus sequence-only), and the *y*-axis is the *q*-value from the two-sided Wilcoxon signed-rank test, with FDR correction performed across motifs, cell types, and insertion distances. A *q*-value threshold of 0.05 is indicated (dashed line). Effects per replicate and insertion distance were normalized by the standard deviation and averaged over three replicates per model type. (C) Box plots of the signed effects of inserting the REST motif 1000 bp upstream of the TSS under each model in non-brain and brain tissues (***: two-sided Wilcoxon signed-rank test, *P *< 1e − 3). (D) Gene expression data for REST across tissue types obtained from the GTEx Portal on 01/23/2026 (https://gtexportal.org/home/gene/REST) (GTEx Consortium 2020).

Transcription factor binding sites are conserved (Nitta *et al.* 2015, Andrews *et al.* 2023). Therefore, we hypothesized that training models with conservation might improve model ascertainment of transcription factor motifs. To test this hypothesis, we interrogated whether models trained with phyloP exhibited improved cell type specific learning of motifs. Specifically, we investigated the effects of motif insertion across CAGE tracks, averaging effects over three replicates of each model (Fig. 4). Multiple motifs were found to have significantly stronger effects in the sequence + phyloP models relative to the sequence-only models, with the REST motif exhibiting the most significant difference (Fig. 4B). REST is a transcription factor that silences neuronal genes in non-neural and neural progenitor tissues (Chen *et al.* 1998). Examining the effect of REST motif insertion in non-neural tissue CAGE tracks revealed that only the sequence + phyloP model learned that the REST motif was associated with gene repression (Fig. 4C). Moreover, the sequence + phyloP model learned that REST-mediated repression was restricted to non-neural tissues, consistent with knowledge of REST biology and expression (Fig. 4C). Similarly, we observed that insertion of the CEBPD motif had significantly larger effects in sequence + phyloP models (Figs. 4B, S7A). The CEBPD, CEBPB, and CEBPE motifs are extremely similar (Fig. S7B), thus we suspect that effects of inserting the CEBPD motif represent effects of all three transcription factors. CEBPB, CEBPD, and CEBPE are highly expressed in immune cells (Fig. S7C) and we observed increased predicted effects of inserting these motifs in immune cell types relative to non-immune tissues for the sequence + phyloP models relative to the sequence-only models. Overall, our motif insertion analysis suggests that incorporating phyloP improves model ascertainment of cell type specific transcription factor effects, which contribute to improved cell type specific expression prediction.

### 3.4 Models trained with or without evolutionary conservation data perform similarly for non-coding variant effect prediction

Non-coding variant effect prediction is an important application of sequence-to-activity models, and we evaluated whether models trained with evolutionary conservation data improve variant effect prediction. To evaluate the ability of models to identify functional non-coding variants, we utilized a dataset of fine-mapped expression quantitative trait loci (eQTLs) from the Genotype-Tissue Expression Consortium (GTEx) (GTEx Consortium 2020) and matched negative variants (Avsec *et al.* 2021). Across six tissues with large eQTL sample sizes, we evaluated eQTL classification performance using predictions from tissue-matched CAGE tracks (Table 3). Interestingly, we found that the sequence-only models slightly outperform the sequence + phyloP models for identifying fine-mapped eQTLs, with the approach of fine-tuning an existing sequence-only model performing similarly to sequence only models. We hypothesized that models trained with phyloP might improve classification of more distal eQTLs; however, we did not see a significant improvment in eQTL classification at any TSS distance by sequence + phyloP models (Table 3).

**Table 3:** eQTL classification performance of sequence-only and sequence + phyloP models trained on cross-species human and mouse data.

Model	phyloP scoring method	**eQTL classification AUROC (matched CAGE track)**				
		All variants	<3 kb	3–12 kb	12–35 kb	35–55 kb
Sequence-only (Kelley 2020)	N/A	0.699	0.708	0.688	0.662	0.634
Sequence-only (retrained)	N/A	**0.702 ± 0.004**	**0.713 ± 0.009**	0.683 ± 0.001	**0.665 ± 0.004**	**0.649 ± 0.002**
Sequence + phyloP	Reference allele phyloP	0.684 ± 0.003	0.699 ± 0.004	0.66 ± 0.005	0.646 ± 0.004	0.639 ± 0.001
	Allele specific phyloP	0.684 ± 0.003	0.699 ± 0.004	0.661 ± 0.005	0.646 ± 0.004	0.639 ± 0.002
	Mask phyloP channel at variant position	0.683 ± 0.003	0.699 ± 0.004	0.66 ± 0.005	0.645 ± 0.005	0.639 ± 0.003
	Mask entire phyloP channel	0.665 ± 0.003	0.686 ± 0.002	0.644 ± 0.002	0.623 ± 0.006	0.627 ± 0.006
Sequence + phyloP finetuned	Reference allele phyloP	0.7 ± 0.001	0.71 ± 0.004	**0.689 ± 0**	0.659 ± 0.002	0.631 ± 0.007
	Allele specific phyloP	0.7 ± 0.001	0.711 ± 0.001	0.687 ± 0.002	0.66 ± 0.001	0.629 ± 0.003
	Mask phyloP channel at variant position	0.7 ± 0	0.712 ± 0.001	0.688 ± 0.001	0.66 ± 0	0.629 ± 0.004
	Mask entire phyloP channel	0.695 ± 0	0.709 ± 0.001	0.681 ± 0.004	0.655 ± 0.004	0.629 ± 0.002

Across six GTEx tissues with large eQTL sample sizes, we evaluated eQTL classification performance using predictions from tissue-matched CAGE tracks. Where possible, we report standard deviations over three model replicates. The highest average AUC in each column is indicated in bold. Results are reported for all variants, as well as stratified by eQTL-gene distance. For the models trained with phyloP, we also compare four different scoring methods: (i) using precomputed phyloP scores based on the human reference genome for both the reference and alternate allele sequences (“Reference allele phyloP”), (ii) recomputing allele specific phyloP scores for each sequence (“Allele specific phyloP”), (iii) masking the phyloP channel at the variant position by setting it to the mean genome-wide phyloP score (“Mask phyloP channel at variant position”), and (iv) masking the phyloP channel across the entire sequence (“Mask entire phyloP channel”).

Finally, we explored different variant scoring methods for models trained with phyloP. Because phyloP is computed using a multiple sequence alignment on the human reference genome, we wondered whether recomputing phyloP scores using the alternate allele would improve variant effect predictions. We evaluated four different scoring methods: (i) using precomputed phyloP scores based on the human reference genome for both the reference and alternate allele sequences, (ii) recomputing allele-specific phyloP scores for each sequence, (iii) masking the phyloP channel at the variant position by setting it to the mean genome-wide phyloP score, and (iv) masking the phyloP channel across the entire sequence. We found that the first three scoring methods yield no significant difference in eQTL classification performance, indicating that the sequence + phyloP models are not relying on the phyloP channel for variant effect prediction. As expected, the final scoring method of masking the phyloP channel across the entire sequence decreases eQTL classification performance, as the inputs are likely significantly out of distribution compared to training sequences.

Overall, our results indicate that models trained with base-pair resolution conservation data do not improve eQTL identification.

## 4 Discussion

In this work, we explored the utility of evolutionary conservation data for improving genomic sequence-to-activity models. We proposed incorporating base-pair resolution phyloP scores as an additional input channel alongside the one-hot encoded DNA sequence to the Basenji2 model. We found that incorporating this conservation metric improves cell type specific sequence-to-expression prediction. By interrogating model attributions, we found that models trained with conservation data better recognize cell type specific regulatory elements, which have been particularly challenging for current sequence-based models to learn (Karollus *et al.* 2023, Kathail *et al.* 2024). Thus, interestingly, although conservation is cell type agnostic, conservation data is most helpful for improving cell type specific gene expression prediction. Somewhat surprisingly, we found that models trained with conservation data do not improve variant effect prediction, providing further evidence of our previous observation that modelling strategies that improve reference sequence prediction do not always improve variant effect prediction (Kathail *et al.* 2024).

We note that even this straightforward method for incorporating conservation data into an existing sequence-to-function model architecture—by including it as an additional input channel—improves cell type specific expression prediction. Determining the optimal strategy for integrating conservation data into neural architectures for sequence-to-function modelling is an interesting direction for future work. Our smoothing ablation results—where models trained with base-pair resolution phyloP outperform models trained on phyloP smoothed to 100 bp resolution—suggest that incorporating conservation data early in the network, before any pooling layers, is likely to yield the greatest benefit. In addition, our results decomposing model attributions into sequence versus phyloP components, as well as examining the TF motif effects learned by sequence-only versus sequence + phyloP models, suggest that a major contribution of phyloP is improved ascertainment of sequence motifs during model training. Therefore, we believe that modeling approaches that allow for learning new or updated sequence motifs are also likely to yield the greatest benefit.

Our fine-tuning results suggest that even with a limited compute budget, an existing sequence-only model can be further improved by augmenting the input sequence with conservation scores. Further work is needed to evaluate whether our results using the Basenji2 model translate to more recent, larger-context models. Although not evaluated in this work, we anticipate that incorporating conservation data may improve model performance for genomes that lack the extensive functional genomics annotations available for the human and mouse genomes.

## 5 Conclusion

In summary, we demonstrated that incorporating conservation into sequence-to-expression models can improve cell type specific expression prediction. Identifying the optimal strategy for incorporating conservation data into neural architectures and exploring the benefit of incorporating additional data modalities—such as human genetic variation (Vitsios *et al.* 2021, Halldorsson *et al.* 2022, Chen *et al.* 2024)—remain interesting directions for future work.

## Supplementary Material

btag626_Supplementary_Data

## Data Availability

All datasets used in this study are publicly available. ENCODE cCREs were downloaded from the SCREEN database (https://screen.encodeproject.org/) (ENCODE Project Consortium 2012). Cell type specific ENCODE cCREs were downloaded from https://encodeproject.org (Table S3). PhyloP scores calculated from the Zoonomia whole-genome alignment of 240 mammalian species were downloaded from https://cgl.gi.ucsc.edu/data/cactus/241-mammalian-2020v2-hub/Homo_sapiens (File name: *241-mammalian-2020v2.bigWig*) (Zoonomia Consortium 2020). The pre-trained Basenji2 model and training data were downloaded from https://console.cloud.google.com/storage/browser/basenji_barnyard (Kelley 2020). For motif insertion analysis, we used position weight matrices for human TFs from the CIS-BP 1.0 database that were downloaded from https://meme-suite.org/meme/db/motifs (Weirauch *et al.* 2014). Fine-mapped eQTL data and matched negative sets were obtained from https://console.cloud.google.com/storage/browser/dm-enformer/data/gtex_fine (Avsec *et al.* 2021). Model training and evaluation code was adapted from the Basenji repository at https://github.com/calico/basenji and the code used in this study is available at https://github.com/ni-lab/basenji-phyloP.
