# Supplementary material for: Base-pair resolution conservation data improves cell type specific sequence-to-expression prediction
Source: Bioinformatics. 2026 Aug 21;42(9):btag626. doi: 10.1093/bioinformatics/btag626 (PMC13574364; doi:10.1093/bioinformatics/btag626)
Supplement: Supplementary file 1 [file btag626_supplementary_data.pdf]

Supplementary Material

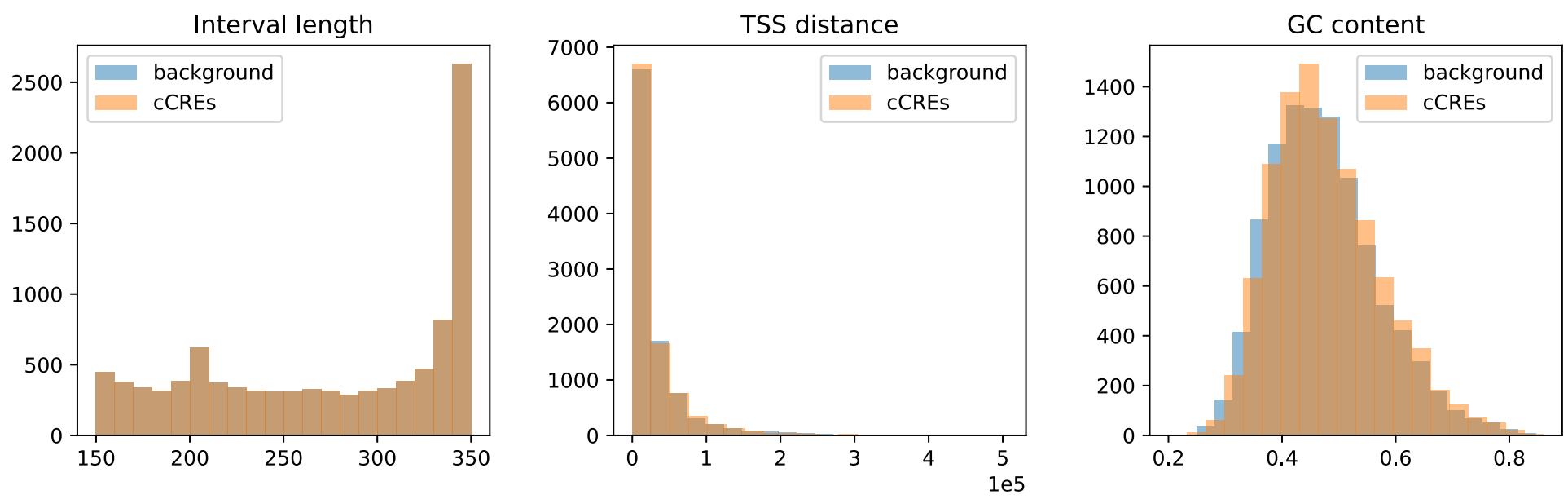

Fig. S1 Genomic attributes of intervals used to measure phyloP. Size (left), TSS distance (middle), and GC content (right) distributions of cCREs and background intervals in Figure 1. For each set of randomly sampled intervals, $N = 10,000$ distinct intervals were sampled.

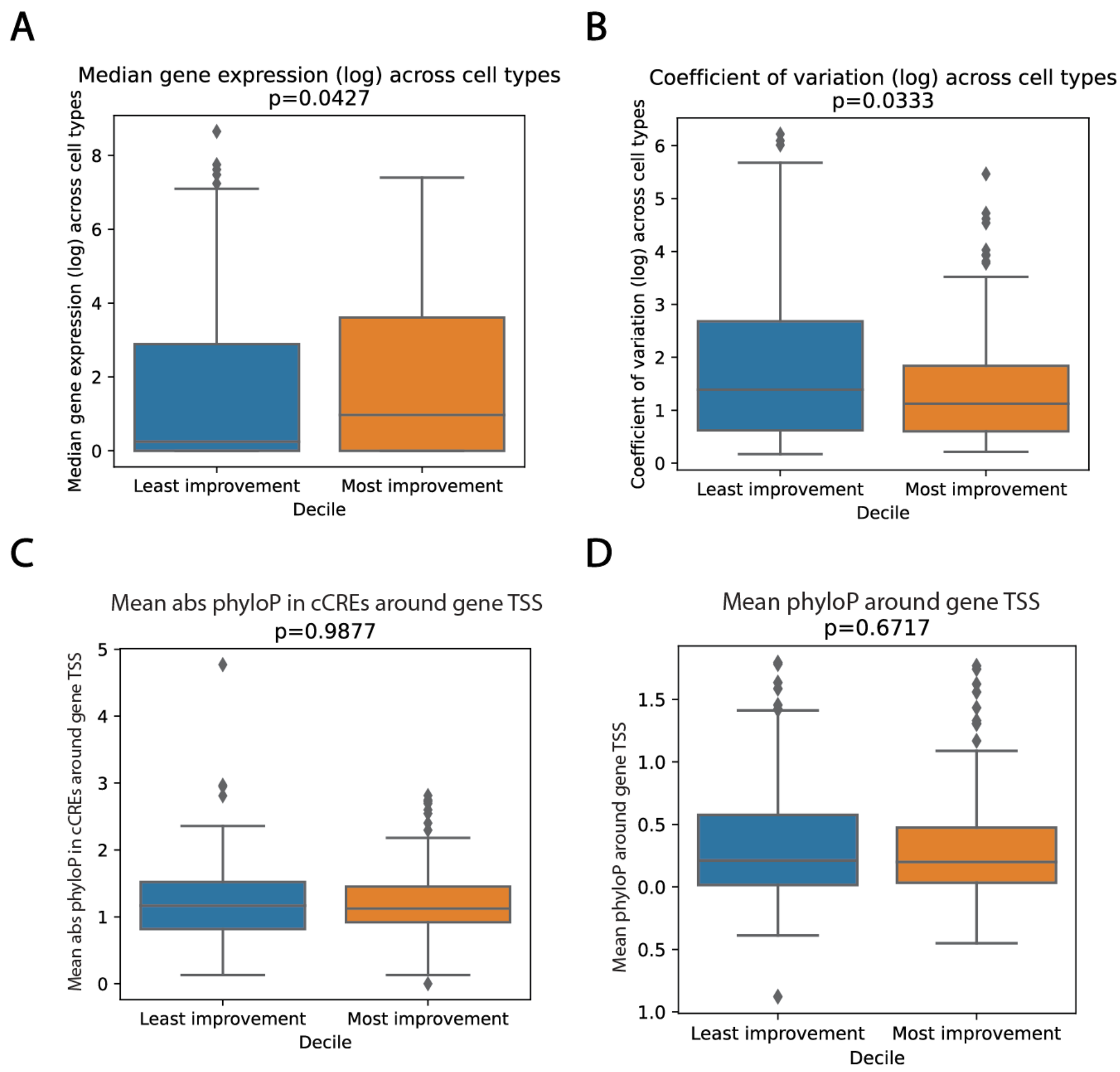

Fig. S2 Additional comparisons of gene properties for the most and least improved genes. Boxplots comparing several gene properties for the top and bottom decile of genes with the highest and lowest improvement in Spearman R across tracks when using sequence + phyloP models. *p*-values are from a two-sided Mann-Whitney U test. The most improved genes are more highly expressed (A) and less cell type specific (B). However, the most improved genes show no trend related to absolute phyloP (which considers both conservation and acceleration signal) within cCREs in a 10kb region around the gene TSS (C), or mean phyloP within the full 10kb region around the gene TSS (D).

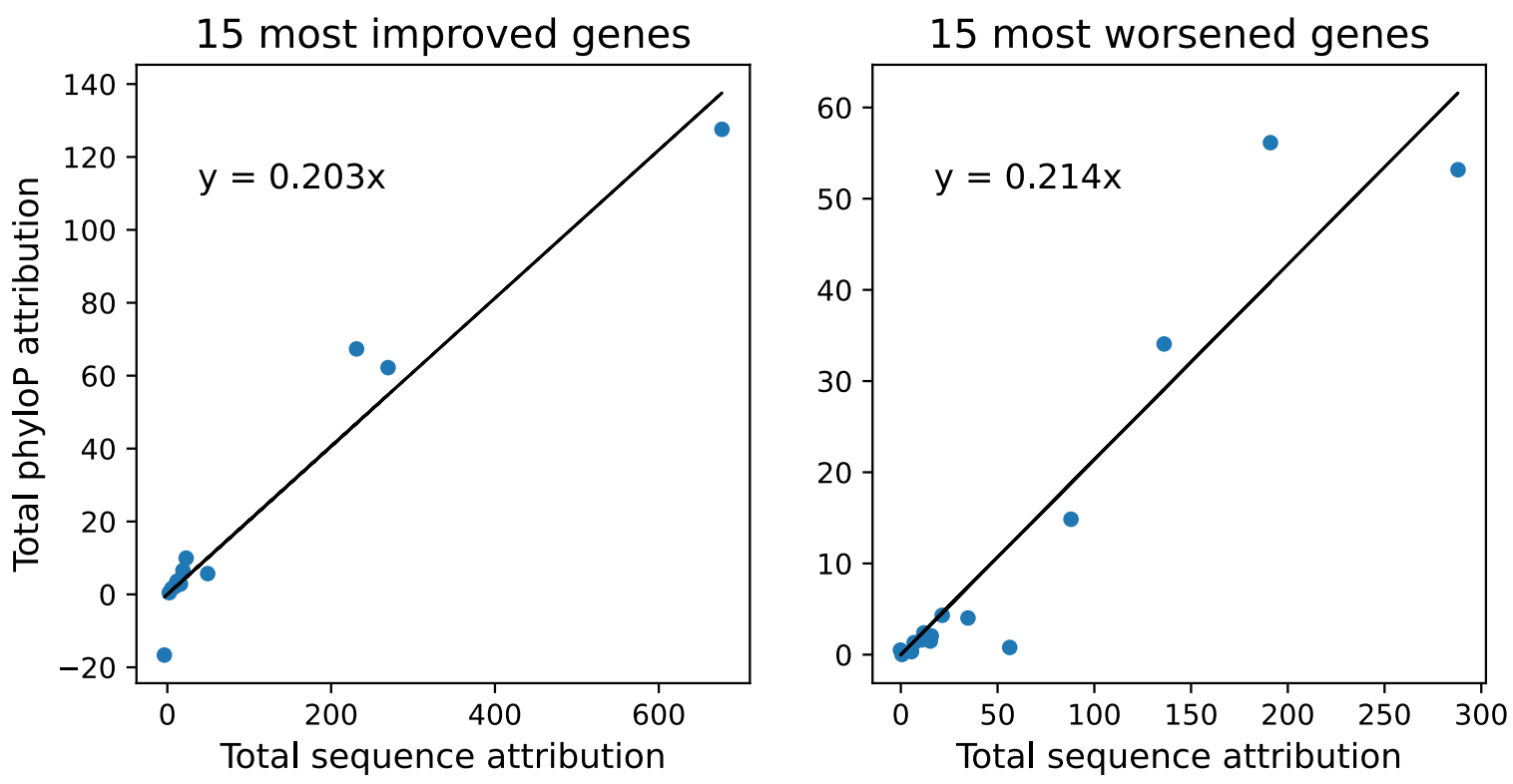

Fig. S3 Total contribution to sequence + phyloP model predictions from phyloP relative to sequence. For the 15 genes whose expression predictions were most improved by the addition of phyloP (left) or most worsened by the addition of phyloP (right), the total phyloP attribution vs total sequence attribution is shown. Total attributions were computed by summing mean CAGE attribution across all input positions for both modalities. The line of best fit assuming an intercept of 0 is shown.

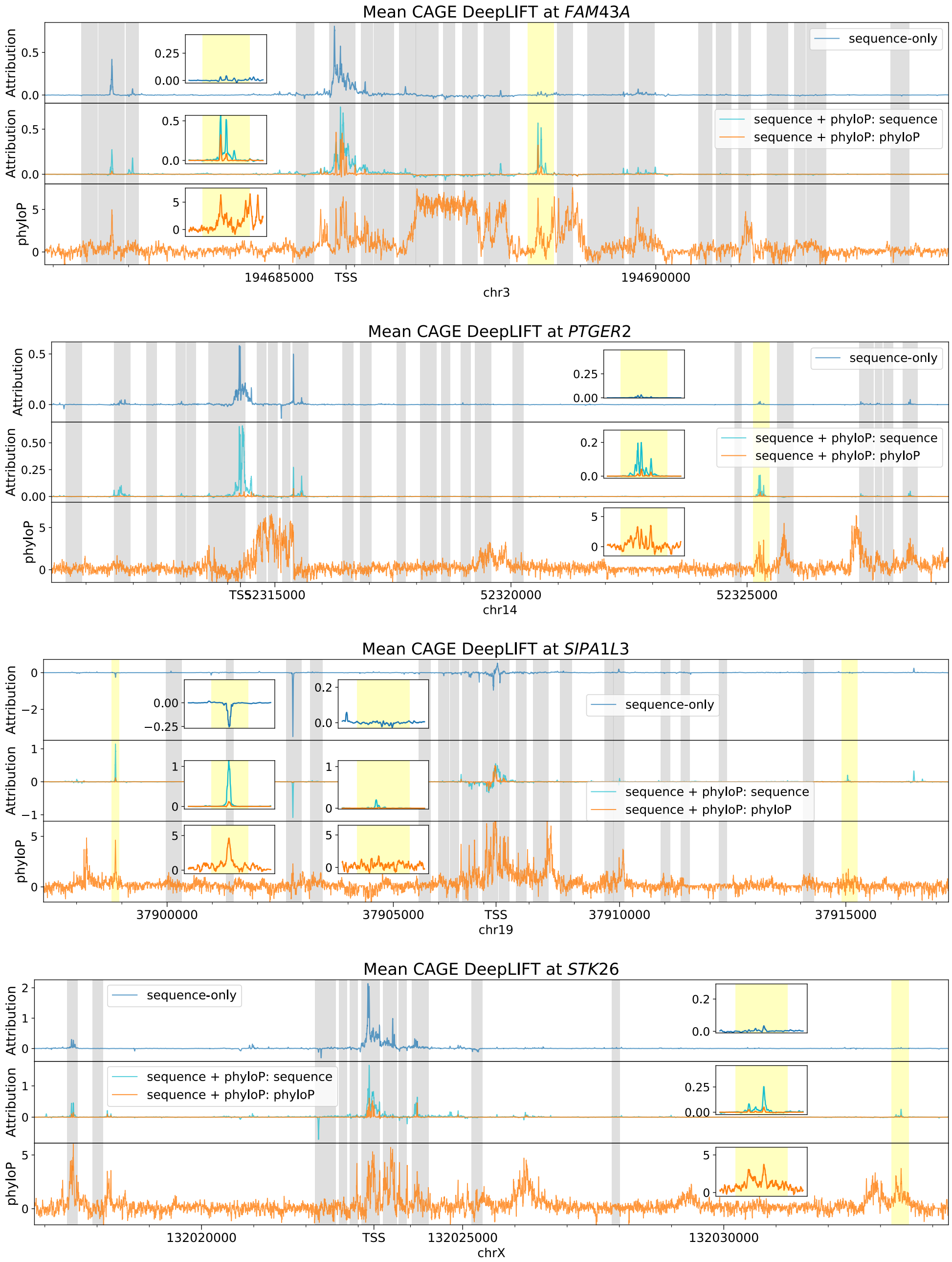

Fig. S4 Attributions at genes where cell type specific expression prediction is reproducibly improved with phyloP. 10bp-window smoothed sequence-only model attributions, sequence + phyloP model attributions, and phyloP scores for four genes (*FAM43A, PTGER2, SIPA1L3, STK26*) among genes with the most improved predictions. Overlapping ENCODE cCREs are highlighted in gray, and selected cCREs are highlighted in yellow and shown in insets.

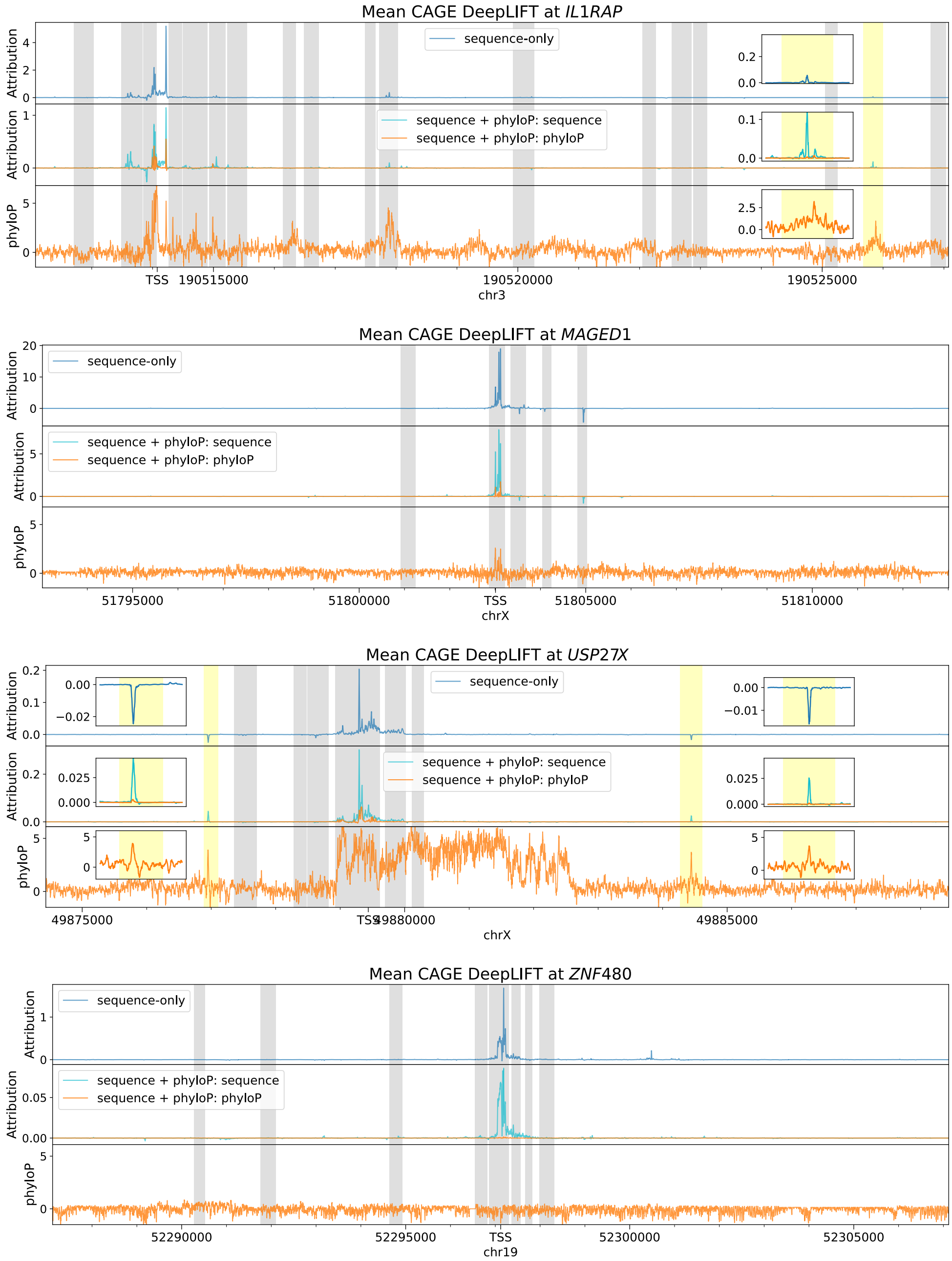

Fig. S5 Attributions at genes where cell type specific expression prediction is reproducibly worsened with phyloP. 10bp-window smoothed sequence-only model attributions, sequence + phyloP model attributions, and phyloP scores for four genes (*IL1RAP, MAGED1, USP27X, ZNF480*) among genes with the most worsened predictions. Overlapping ENCODE cCREs are highlighted in gray, and selected cCREs are highlighted in yellow and shown in insets.

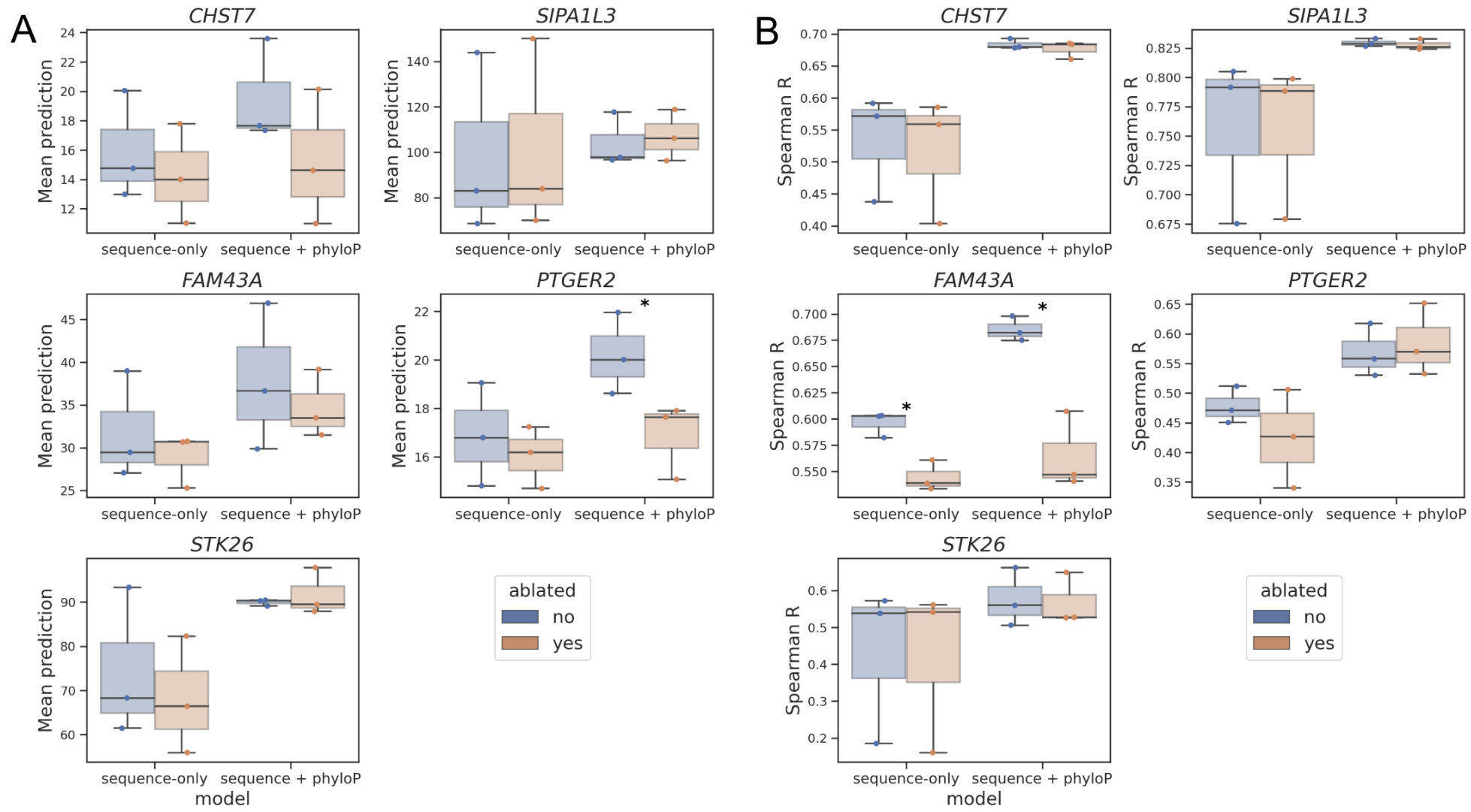

Fig. S6 Results of ablating highlighted yellow cCREs in genes whose cell type specific expression predictions are improved by phyloP. For previously shown attribution loci representing genes whose predictions were reproducibly improved by phyloP (Figure 2C, Figure S4), the yellow highlighted cCREs with differential attributions were ablated by replacing the sequence in those regions with the unknown nucleotide (N) and phyloP with the genomic phyloP mean. Each model was run on the unablated and ablated inputs to predict CAGE expression at the TSS. $*$: two-sided t test, $p < 0.1$. (A) Mean prediction across CAGE tracks of each model replicate. (B) Spearman R across CAGE tracks of each model replicate.

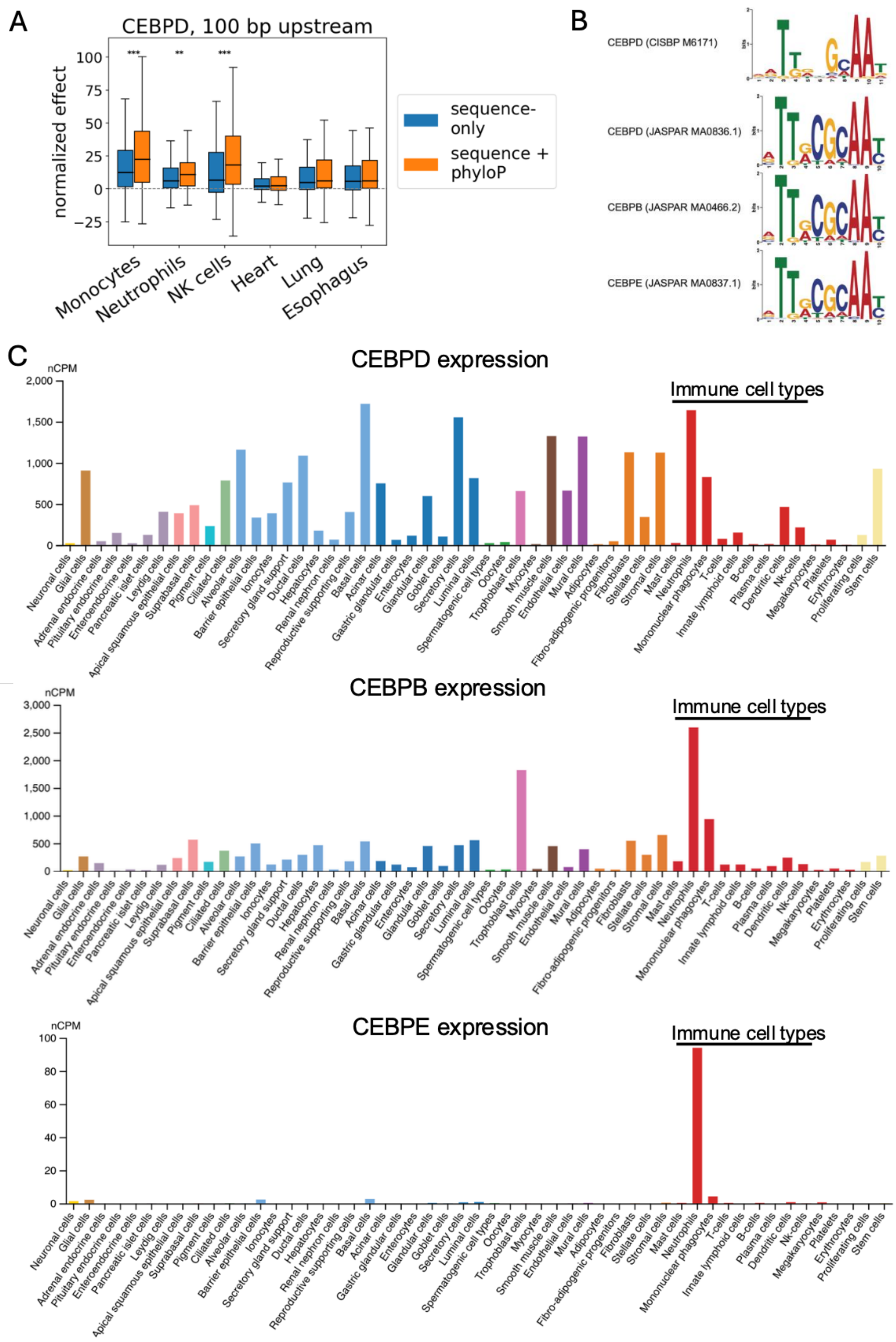

Fig. S7 Differential learning of CEBPD motif. (A) Boxplots of the signed effects of inserting the CEBPD motif 100 bp upstream of the TSS in immune and non-immune cell types. Two-sided Wilcoxon signed-rank test, ***: $p < 0.001$, **: $p < 0.01$. (B) Alignment of CEBP motifs. (C) Publicly available gene expression data for CEBPD (https://www.proteinatlas.org/ENSG00000221869-CEBPD/summary/rna), CEBPB (https://www.proteinatlas.org/ENSG00000172216-CEBPB/summary/rna), and CEBPE (https://www.proteinatlas.org/ENSG00000092067-CEBPE/summary/rna) across cell types .

Dataset	Used for	Description
Basenji2 training data (Kelley, 2020)	Model training (outputs)	ATAC-seq, DNase-seq, ChIP-seq, and CAGE-seq for 5,313 human and 1,642 mouse tracks.
phyloP (Zoonomia Consortium, 2020)	Model training (augmented inputs)	Base-pair resolution conservation and acceleration scores calculated from the Zoonomia whole-genome alignment of 240 mammalian species.
Cell type specific cCREs (ENCODE Project Consortium, 2012)	Evaluation (cell type specific attributions)	Candidate *cis* regulatory elements determined by the ENCODE project.
TF motif database (Weirauch et al., 2014)	Evaluation (TF motif activity)	Position weight matrices for human TFs from the CIS-BP 1.0 database.
eQTLs (GTEx Consortium, 2020; Avsec et al., 2021)	Evaluation (variant effect prediction)	Fine-mapped eQTLs and matched negative variants from GTEx.

Table S1 Description of datasets used for training and evaluation.

Model	Dataset	Test Pearson R	Test Spearman R
sequence-only	expressed TSSs	0.473	0.487
phyloP channel	expressed TSSs	0.486	0.504
phyloP multiply $d = 1$	expressed TSSs	0.463	0.477
phyloP multiply $d = 450$	expressed TSSs	0.492	0.488
sequence-only	Genomic tiling	0.278	0.151
phyloP channel	Genomic tiling	0.438	0.214
phyloP multiply $d = 1$	Genomic tiling	0.431	0.205
phyloP multiply $d = 450$	Genomic tiling	0.434	0.207

Table S2 Testing alternative approaches of incorporating phyloP. Here, we trained a DeeperDeepSEA architecture (Chen et al., 2019) on the single task of predicting K562 CAGE signal, either with only sequence as input, with an added phyloP input channel, or by applying a sigmoidal transformation to phyloP base-pair level scores and multiplying the output of the second convolutional layer by these scores. We learned either a single sigmoidal transformation for all output channels ($d = 1$), or a different sigmoidal transformation per output channel ($d = 450$).

**cCREs URL**	**cCREs description**
https://www.encodeproject.org/files/ENCFF347BXP/	fibroblast of pulmonary artery
https://www.encodeproject.org/files/ENCFF668RON/	neuronal stem cell
https://www.encodeproject.org/files/ENCFF243PZI/	epithelial cell of proximal tubule
https://www.encodeproject.org/files/ENCFF398LPB/	neutrophil
https://www.encodeproject.org/files/ENCFF461WTM/	skeletal muscle cell
https://www.encodeproject.org/files/ENCFF359NDM/	smooth muscle cell
https://www.encodeproject.org/files/ENCFF770UDU/	hepatocyte
https://www.encodeproject.org/files/ENCFF347CZR/	neuron
https://www.encodeproject.org/files/ENCFF421AOR/	epidermal melanocyte
https://www.encodeproject.org/files/ENCFF714ECJ/	endothelial cell of umbilical vein

Table S3 List of URLs and descriptions for cell type specific cCREs.
